# Targeting cytoskeletal phosphorylation in cancer

**DOI:** 10.37349/etat.2021.00047

**Published:** 2021-06-28

**Authors:** Clara Llorente-González, Marta González-Rodríguez, Miguel Vicente-Manzanares

**Affiliations:** Molecular Mechanisms Program, Centro de Investigación del Cáncer and Instituto de Biología Molecular y Celular del Cáncer, Consejo Superior de Investigaciones Científicas (CSIC)-University of Salamanca, 37007 Salamanca, Spain; Changchun Institute of Applied Chemistry, Chinese Academy of Sciences, China

**Keywords:** Cytoskeleton, phosphorylation, cancer, actin, tubulin, vimentin, myosin

## Abstract

Phosphorylation of cytoskeletal proteins regulates the dynamics of polymerization, stability, and disassembly of the different types of cytoskeletal polymers. These control the ability of cells to migrate and divide. Mutations and alterations of the expression levels of multiple protein kinases are hallmarks of most forms of cancer. Thus, altered phosphorylation of cytoskeletal proteins is observed in most cancer cells. These alterations potentially control the ability of cancer cells to divide, invade and form distal metastasis. This review highlights the emergent role of phosphorylation in the control of the function of the different cytoskeletal polymers in cancer cells. It also addresses the potential effect of targeted inhibitors in the normalization of cytoskeletal function.

## Introduction

Cytoskeletal proteins form the backbone of the different types of structural polymers found in every eukaryotic cell. Such polymers include microfilaments (MF), mini-filaments, microtubules (MT) and intermediate filaments (IF). Each polymer has a relatively homogeneous composition. Monomeric cytoskeletal proteins bind in a head-to-tail manner to form long chains of different geometries and biophysical properties. These monomers include actin (which forms MF), myosin (mini-filaments), tubulin (MT), and various families of IF proteins, including keratins, desmins, glial fibrillary acidic protein (GFAP), peripherin, vimentin, internexins, nestins and others (reviewed in [1]). MFs and mini-filaments enable cells to adapt to their surroundings. They exert several roles in cell division and support cell migration in physiological and pathological contexts, for example during invasion and metastasis. MTs are essential as they form the physical scaffold that mediates an even separation of genetic material during cell division, but they play limited roles in cell migration. IFs confer mechanical resistance to the cells.

Like every protein in the eukaryotic proteome, cytoskeletal proteins are substrates of diverse protein kinases. Phosphorylation changes their interactive and dynamic properties with respect to their non-phosphorylated forms. In the specific case of cytoskeletal proteins, phosphorylation controls their assembly and disassembly affinity and dynamics, as well as the biochemical and biophysical properties of the polymers themselves. Phosphorylation also modulates their interactome, which also affects the stability and function of the polymers.

Genetic modifications affecting protein kinases are very frequent in cancer [2]. The most typical are mutations or deletions that cause loss of function or increased catalysis [3]. These two outcomes also emerge when portions of kinases become fused with incorrect pieces of DNA as part of genomic cancer recombination, leading to abnormal activation. One example is the Philadelphia translocation, which is typical of chronic myeloid leukemia (CML) cells. It consists of a reciprocal translocation between parts of human chromosomes 9 and 22 that leads to the production of a constitutively active Abelson kinase (BCR-ABL), which triggers uncontrolled proliferation by phosphorylating multiple substrates [4]. On the other hand, activating mutations may lead to unexpected effects on the different cytoskeletal systems. For example, mutations to the small GTPase RhoA may lead to increased activation of proteins that control mini-filament formation [5]. These events disturb the delicate balance of MFs and mini-filament dynamics that enable cells to maintain their form and function in the context of the host tissue.

These and other examples found in the latter sections of the present work highlight the fact that alterations of the phosphorylation of cytoskeletal proteins caused by cancer-related mutations may change the dynamics, architecture, and function of the different cytoskeletal polymers. These events cause aberrant molecular behaviors that may confer specific properties to cancer cells, e.g., increased cell migration, invasion, division, mechanosensing, etc.

The study of these modifications is complicated by the existence of multiple isoforms of these proteins, some of which have non-overlapping functions. There are many isoforms of actin, tubulin and myosin, and multiple forms and variants of IF. How phosphorylation impacts every isoform of every cytoskeletal protein is not only impossible to describe, but mostly unknown. Because of this, we describe only the current state of the art regarding the phosphorylation of selected isoforms of actin, myosin II, tubulin and vimentin. We focus on the functional effect of these phosphorylations, and what the consequences would be if the extent of these phosphorylations was altered in the context of cancer progression. While phosphorylation has proven crucial for the function of some of these filament-forming polymers, e.g., myosin II, the function of many of the phosphorylations described here is still unexplored. Thus, a major goal of this work is to provide a wide, yet incomplete, perspective of the field, identifying potential hotspots that may be amenable to specific targeting to treat diverse forms of cancer.

## Actin

Actin forms MFs by adenosine triphosphate (ATP)-dependent polymerization. Together with myosin II mini-filaments, MFs constitute the contractile apparatus of animal cells. A vast array of nucleators, cross-linkers and other binding partners regulate multiple aspects of its ability to form filaments and the multiple cellular functions they enable [6, 7].

**Figure 1.**
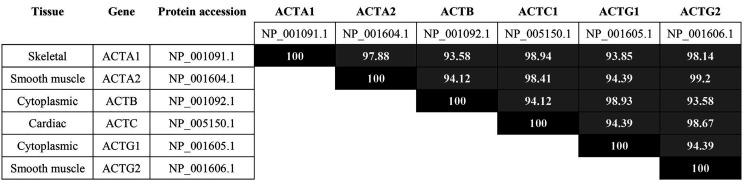
Homology between human actin isoforms

There are multiple isoforms of actin, including muscle-specific and non-muscle [8]. Due to its abundance, ubiquity and high degree of homology among isoforms (Figure 1), we focus on cytoplasmic, β-actin. Human β-actin (ACTB gene; Uniprot #P60709) is located in chromosome 7p22.1 in humans. β-actin forms MFs in every non-muscle cell lineage, mediating protrusion, assembly of contractile structures and other motility-related structures, e.g., podosomes [9]. Somatic mutations of the ACTB gene associated to cancer have not been reported. However, the filamentous state of actin is a checkpoint for cell proliferation that is deregulated in several types of cancer [10]. Although the effect of actin phosphorylation in MF dynamics has yet to be studied in detail, some phosphorylation events have been described that potentially affect actin filamentation and/or disassembly (see Table 1 for a list). For example, serine (Ser)33 appears phosphorylated in multiple phospho-proteomic analyses, including diverse types of cancer cells that include human epidermal growth factor receptor 2 (HER2)-positive, luminal A and triple negative breast cancer [2, 11] and lung cancer (http://phosphosite.org, search term = ACTB, Ser33). Its phosphorylation lies downstream of polo-like kinase 1 (PLK1) since the specific inhibitor BI 4834 abrogates it [12]. Although it is unclear whether PLK1 directly phosphorylates β-actin in Ser33, the crucial role of this kinase in cancer cell division [13] suggests that PLK1-dependent actin phosphorylation may control actin function (by controlling actin polymerization and/or cross-linking) in cancer cell proliferation.

**Table 1.** Main human β-actin phosphorylation sites

**Gene**	**Site**	**Putative kinase**	**Discovered by/inhibitor**	**Effect**	**References**
*ACTB*	Ser33	PLK1	BI_4834	Probably mitosis	[12]
	Tyr53	Src	Biochemical assays, targeted mutation	MF disassembly	[16, 17]
	Tyr91	EGFR	erlotinib	not known	[27]

EGFR: epidermal growth factor receptor; Tyr: Tyrosine

Another potentially important residue is Tyr53. It is conserved from *Dictyostelium discoideum* to humans. It resides in the D-loop of actin [14], which is essential for actin polymerization [15]. Tyr53 phosphorylation decreases the affinity of actin monomers for each other, causing filament shortening [16]. This mechanism is critically important in the central nervous system. During synaptogenesis, Tyr53 phosphorylation increased actin turnover [17]. Although the mitogen-activated protein kinase kinase (MEK) inhibitor U0126 inhibits its phosphorylation (and that of Tyr362, of unknown function), it is unlikely that this kinase, which phosphorylates Ser/threonine 18 (Thr), directly phosphorylates Tyr53 (or Tyr362). However, MEK control, directly or indirectly, some potential Tyr kinases that may phosphorylate Tyr53 (and/or Tyr362). For example, extracellular signal-regulated kinase (ERK), the canonical target of MEK [18], can phosphorylate and activate RhoA [19], which increases focal adhesion maturation [20]. In this manner, ERK would increase Src activation by recruiting it to focal adhesions. However, active Src does not remain in focal adhesions, but propagates rapidly [21]. Based on these data, a possible model emerges in which Src is activated at focal adhesions in a MEK/ERK-dependent manner. Active Src would diffuse from focal adhesions and phosphorylate filamentous actin in Tyr53. This could destabilize MFs, thereby decreasing its assembly in contractile actomyosin bundles associated to focal adhesions. According to the actin treadmilling model [7], monomeric actin would eventually undergo Tyr53 dephosphorylation to be reused by the cell to form other structures, e.g., lamellipodia or podosomes/invadopodia in less contractile/more protrusive regions. In this regard, Src overexpression promotes invadopodia [22] which, similar to podosomes, appear in regions in which actomyosin bundles are scarce. Importantly, Tyr53 also appears nitrated [23], which accelerated filament elongation, promoting the formation of disorganized F-actin aggregates, which may be very important for actin dynamics in highly oxidative contexts, e.g., in lung cancer [24].

Finally, actin phosphorylation in Tyr91 has been observed in multiple types of cancer, including diverse subtypes of breast cancer [2], colorectal carcinoma [25], lung cancer [26] and diverse types of leukemia (http://phosphosite.org, search term = ACTB, Tyr91). Tyr91 phosphorylation was modestly affected by treatment of non-small cell lung cancer cells with the EGFR inhibitor erlotinib [27]. However, the effect of this phosphorylation in the regulation of the actin cytoskeleton in cancer cells has yet to be addressed, although it could be related to its ability to polymerize and/or form filaments. Similar to Tyr53, Tyr91 also appears nitrated *in vivo* [23], with potential implications in the regulation of actin dynamics by the oxidative state of the cell.

## Non-muscle myosin II

Functionally, non-muscle myosin II (NMII) is a hexameric molecular motor made of different combinations of genes. It always comprises two heavy chains [myosin heavy chain II (MHCII)] and four light chains, two regulatory (RLC) and two structural (essential, ELC). There are three genes that encode MHCII isoforms: MYH9 (Uniprot #P35579, human chromosome 22q12.3), MYH10 (Uniprot #P35580, human chromosome 17p13.1) and MYH14 (Uniprot #Q7Z406, human chromosome 19q13.33); three genes that encode RLC: MYL9 (Uniprot #P24844, human chromosome 20q11.23), MYL12A (Uniprot #P19105, human chromosome 18p11.31) and MYL12B (Uniprot #O14950, human chromosome 18p11.31); and one gene that encodes ELC, MYL6 (Uniprot #P60660, human chromosome 12q13.2). The typical structure of NMII involves MHCII from the same gene forming a central homodimer (they do not heterodimerize). Each heavy chain contains two tandem IQ motifs that bind to ELC and RLC. These binding sites define the “neck” of the hexamer, which is flexible and enables the conformational movement that generates mechanical work upon ATP hydrolysis when the hexamer is bound to actin [28]. Actin binding and ATPase activities lie upstream of the neck in a ≈ 800 amino acid long globular head domain. Downstream of the neck, both heavy chains display a ≈ 1,000 amino acid long coiled-coil domain that supports dimerization. The C-terminus of the heavy chains ends in a non-helical domain of variable length that controls the oligomerization of the hexamer into larger order units termed mini-filaments (the name has a historic connotation based on electron microscopy (EM) visualization of thick and thin bands in muscle sarcomeres, which are made of myosin II and actin, respectively).

Whereas binding to calcium-sensitive proteins controls muscle myosin II function, NMII is largely controlled by phosphorylation. In fact, RLC phosphorylation is essential for the conformational extension that is required for NMII hexamers to form mini-filaments [29]. Likewise, phosphorylations in the coiled coil domain controls dimerization; and those in the non-helical tailpiece (NHT) domain regulate oligomerization [30]. Phosphorylations of the globular domain of the heavy chain are less characterized. In Table 2, it summarizes the phosphorylations affecting the different chains of the NMII hexamer, including phosphorylations of RLC (MYL9/12) that modulate its function as well as that of the entire NMII hexamer; those of ELC (MYL6); as well as of the three genes of MHCII (*MYH9/10/14*).

**Table 2.** Main human NMII (RLC, ELC and MHCII-A, B, C) phosphorylation sites

**Gene**	**Site**	**Putative kinase**	**Discovered by/inhibitor**	**Effect**	**References**
*MYL9/MYL12*	Ser1/2	PKCα	Targeted mutation	Inhibits ATPase activity	[32, 33]
	Thr18	CITK, ZIPK, ROCK1/2	Targeted mutation, biochemical assays	Synergizes with pSer19 to stabilize conformation and boost ATPase activity	[38, 109]
	Ser19	MLCK, MRCK, CITK, ZIPK, ROCK1/2	ML-7, dominant negatives, Y-27632, siRNA	Conformational extension and increased ATPase activity	[35, 110]
	Tyr155	EGFR	Targeted mutation, cetuximab	Inhibited NMII assembly	[37]
*MYL6*	Tyr29	not known	not known	Carcinoma progression	[111]
	Tyr89	EGFR?	Genfitinib	not known	[41]
*MYH9*	Tyr158	Src	siRNA	Decreases listeria infection	[112]
	Thr1800, Ser1803, Ser1808	TRPM6/7	Biochemical assays	Decreases filament stability	[43]
	Ser1916	PKCβ	Go6976	Decreases filament stability, increases Mts1 binding	[45]
	Ser1943	CK-II	Targeted mutation	Decreases filament formation	[47]
*MYH10*	Ser1810, Thr1815	TRPM6/7	Biochemical assays	Decreases filament stability	[43]
	Ser1935	PKCζ	Targeted mutation, PKCζ pseudosubstrate	Impairs filament stability and cell polarity	[49]
	Ser1937	PKCζ	Biochemical assays, siRNA	Impairs filament stability	[48]
*MYH14*	Thr1832/1838	TRPM6/7	Biochemical assays	Decreases filament stability	[43]

PKCs: protein kinase C; ROCK: RhoA-coiled coil kinase; MLCK: myosin light chain kinase; CITK: citron kinase; MRCK: myotonic dystrophy kinase-related; ZIPK: zipper-interacting protein kinase; siRNA: small interfering RNA; TRPM6/7: transient receptor potential melastatin 6/7

RLC is the most important regulatory hotspot of myosin II by phosphorylation. Several residues have been described, including Ser1/2. Their phosphorylation inhibits NMII function, as they decrease ATP catalysis on the NMII hexamer head [31]. Although they control NMII function in response to growth factors downstream of conventional PKCs [32, 33], their mutation to a non-phosphorylatable version does not prevent cell division [34].

Conversely, Ser19 phosphorylation mediates the conversion of folded, assembly incompetent into extended, assembly competent NMII hexamers. Extended hexamers whose RLC is phosphorylated in Ser19 immediately form bipolar filaments that grow by lateral association, as outlined below [29]. Ser19 phosphorylation also increases ATP catalysis in the associated MHCII [35, 36]. On the other hand, Thr18 only appears phosphorylated if Ser19 is also phosphorylated [37]. Based on its *in vitro* effect boosting ATP catalysis of the bound MHCII, Thr18 is considered a synergy site with Ser19. It also has increases the half-life of NMII mini-filaments, which is essential during cell migration [38]. Several kinases induce these phosphorylations, including ROCK, MLCK, CITK, MRCK and ZIPK/death-associated protein kinase 3 (DAPK3) [30].

Very recently, we have identified Tyr155 phosphorylation downstream of EGFR. However, this phosphorylation only occurs when RLC is not bound to NMII. Tyr155 phosphorylation prevents the association of RLC with NMII, thus de facto decreasing the amount of NMII available to form filaments [37].

On the other hand, the role of ELC phosphorylation in cellular physiology or NMII function remains practically unexplored. Tyr29 appears more phosphorylated in many types of cancer, but the kinase remains unidentified [39]. Conversely, Tyr89 phosphorylation is dependent of EGFR III [40] and its phosphorylation is inhibited in cells treated with the EGFR inhibitor gefitinib [41]. However, whether this phosphorylation decreases NMII assembly is unknown, and currently under investigation in our lab.

Regarding the heavy chains, there are isoform-specific differences regarding heavy chain phosphorylation that have potential effects on diverse types of filaments depending on their molecular composition. In general, head domain phosphorylations have the potential to control actin binding and ATPase activity of myosin II. However, this has been poorly explored. Conversely, phosphorylations of the coiled coil domains of MHCII-A/B/C are better characterized. These regions are important for dimerization, and phosphorylation has been shown to decrease the stability of the hexamers and hinders their lateral association with other hexamers to form filaments. This revealed that lateral interactions are highly dependent on the net charge of the interacting regions [42]. Phosphorylation of Thr1800, Ser1803 and Ser1808 in MHCII-A, Ser1810 and Thr1815 in MHCII-B and Thr1832/Ser1838 in MHCII-C decrease the formation of filaments of the corresponding isoform [43]. The kinases that regulate most of these phosphorylations are α-kinases, for example TRPM6/7 [43]. However, how theses kinases distinguish between isoforms is unclear.

Finally, phosphorylations at the end of the coiled-coil or into the NHT domain of MHCII decrease mini-filament formation. One such residue is Ser1916 in MHCII-A. Its phosphorylation by PKCβ reduces filament stability [44, 45] as it increases NMII-A interaction with Mts1/S100A4, which forces NMII-A to stay in an assembly-incompetent conformation [46]. Phosphorylation of Ser1943 in MHCII-A has this effect. Casein kinase (CK)-II phosphorylates MHCII-A in Ser1943, promoting NMII-A filament disassembly [47]. In MHCII-B, Ser1935 and Ser1937 (MHCII-B) are phosphorylated by PKCζ [48, 49], but they haveasimilareffect. Importantly, and because NMII-B filaments are more stable than those made of NMII-A, these phosphorylations impair cell polarity and migration [49].

## Tubulin

**Figure 2.**
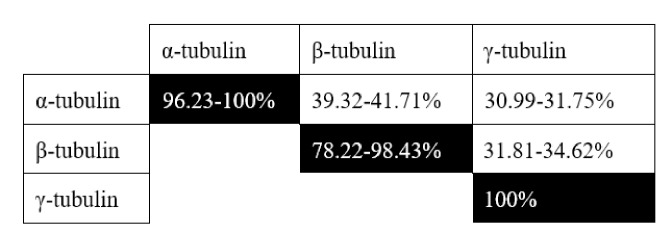
Grouped homology among human tubulin isoforms. Range shown in the homology of an isoform with itself refers to the minimal and maximal homology among sub-isoforms (see Table S1 for full details)

There are three isoforms of tubulin, each one including several variants. For the sake of brevity, and due to the grouped homology among them (Figure 2 and Table S1), we focus on α1-tubulin, encoded by the gene TUBA1A (Uniprot #Q71U36, human chromosome 12q13.12), β1-tubulin, encoded by the gene TUBB1 (Uniprot #Q9H4B7, human chromosome 20q13.32) and γ1-tubulin, encoded by the gene TUBG1 (Uniprot #P23258, human chromosome 17q21.2). α- and β-tubulins are the major components of polymeric MTs. γ-tubulin is mainly present at the centrosome [also known as MT-organizing center, (MTOC)], nucleating polymerization by forming the γ-tubulin ring complex (γTuRC). This complex acts as a template for α/β monomer incorporation [50]. Phosphorylations affecting these subunits are summarized in Table 3, but their effects in tubulin dynamics are very poorly characterized, particularly in cancer. A few significant residues include Ser48 (and Ser75 of β-tubulin). These are likely Aurora kinase sites, as two independent Aurora kinase (AURK) inhibitors reduce their phosphorylation levels [51]. Due to the key role of AURK in cancer [52], it will be extremely interesting to study whether these sites are involved in the potential regulation of MT dynamics. In this regard, Aurora kinase A (AURKA) inhibition inhibits osteosarcoma cell division by preventing MT stabilization to form the mitotic spindle [53].

**Table 3.** Main human tubulin phosphorylation sites

**Gene**	**Site**	**Putative kinase**	**Discovered by/inhibitor**	**Effect**	**Reference**
*TUBA*	Ser48	AURK	AZD1152, ZM447439	Not known	[51]
	Ser165	PKCα	bisindolylmaleimide	EMT	[54]
*TUBB*	Ser75	AURK	AZD1152, ZM447439	Not known	[51]
*TUBG*	Ser131	SadB	Targeted mutation	Centrosome duplication	[55]
	Ser385	SadB	Targeted mutation	Promotes γ-tubulin interaction with the chromatin	[56]

SadB: synapses of amphids defective kinase

On the other hand, tubulin phosphorylation in Ser165 by PKCα increases MT dynamics, cell motility and acquisition of a mesenchymal phenotype characterized by expression of neural (N)-cadherin [54]. This strongly suggests that this phosphorylation could be a key event in the acquisition of mesenchymal phenotypes, which is a typical event during the transition of tumors from non-invasive to invasive states.

Finally, phosphorylation of centrosomal γ-tubulin in Ser131/385 is mediated by SadB. This phosphorylation is involved in centrosome duplication during mitosis, a key event during cell division. A phospho-mimetic form induces spontaneous centrosome duplication, whereas a non-phosphorylatable mutant impairs centrosome duplication [55]. SadB also phosphorylates γ-tubulin in Ser385, regulating S-phase progression by moderating the activities of E2 promoter-binding factors. When phosphorylated by SadB in this amino acid, γ-tubulin increases its nuclear localization [56].

## Vimentin

Vimentin (UniProt # P08670) is located on human chromosome 10p13. Vimentin forms very stable IF. Unlike actin, myosin and tubulin, IFs are not very dynamic, but they endow cells with structural stability. Importantly, vimentin is an IF typical of mesenchymal and hematopoietic cells, and a signature gene of the epithelial-mesenchymal transition (EMT) [57]. As such, it is a marker of cells that evolve into mesenchymal phenotypes, acquiring migratory capability as tumors become invasive. The development of cancer affects this protein, including epigenomic alterations [58, 59] and somatic mutations in squamous lung cancer [60], gastric adenocarcinoma [61], and other types of cancer.

Vimentin undergoes extensive phosphorylation, which potentially controls its cellular function. The best characterized phospho-residues are grouped in the non-helical *N*-terminus domain [62] (see Table 4). Some crucial residues include Ser5, Ser7, Ser8, Ser9 and Ser10, which are phosphorylated by PKCα [63]. Since phosphorylation of these residues control leukocyte transmigration downstream of phosphatidyl inositol 3-kinase, isoform γ (PI3Kγ) [64], it is possible that they also control CTC extravasation, which tends to mimic leukocyte diapedesis [65]. In this regard, vimentin localizes to the trailing edge of migrating leukocytes and controls cortex rigidity [66]. This may be important to reduce cancer cell attrition in the bloodstream [67]. The ras-related C3 botulinum toxin substrate 1 (Rac1)/p21-activated kinase 1 (PAK1) pathway controls the phosphorylation of Ser26, Ser51 and Ser66, impairing IF assembly. Whether this is related to the weak oncogenic ability of Rac [68] is currently unknown.

**Table 4.** Main human vimentin phosphorylation sites

**Gene**	**Site**	**Putative kinase**	**Discovered by/inhibitor**	**Effect**	**Reference**
*VIM*	Ser5/7/8/9/10	PKCα	Calyculin A (phosphatase)	Cell polarity	[63]
	Ser26/51/66	PAK1	Biochemical assays, selumetinib, vemurafenib	Abnormal assembly	[113, 114]
	Ser39	AKT/PKB	A-674563	Proteolytic protection, slowed polymerization?	[69]
	Ser56	AKT/PKB ROCK	A-674563 siRNA	Filament disassembly	[69] [72]
	Ser72	ROCK1	Targeted mutation, Y27632	Increased cell migration	[75, 76, 115]
	Ser73	AURKB	Biochemical assays	Mitosis?	[114, 116]
	Ser83	PLK-1, CaMKII	Biochemical assays, KN-93 (CaMKII inhibitor)	Filament disassembly and pathogen interaction	[77, 117, 118]

CaMKII: calmodulin-dependent protein kinase II

Key residues include Ser39, Ser56, Ser72 and Ser83. In cancer cells, Ser39 phosphorylation by protein kinase B (AKT/PKB) protects vimentin from proteolysis and enhances tumor growth and metastasis [69] by altering filament assembly [63], which regulates cortex plasticity and could underlie the fact that cancer cells are overall softer than non-cancer cells [70].

On the other hand, Ser56 is phosphorylated by the cyclin dependent kinase (CDK) 1. This phosphorylation recruits PLK1, which enhances phosphorylation in Ser82 [71]. Consequently, cell arrest in G2/M induced by taxanes lead to an accumulation of phospho-Ser56 vimentin in a CDK1-dependent manner. Ser56 is also phosphorylated downstream of ROCK and PAK1 in hypoxia [72]. Ser56 phosphorylation promotes disassembly of perinuclear vimentin, controlling filament stability [73] and promoting cancer cell invasiveness [74]. Likewise, two independent studies indicated that Ser72 phosphorylation downstream of ROCK1 is important for cancer cell migration. A phospho-mimetic mutation of Ser72 increased cancer cell speed, whereas a non-phosphorylatable form impaired sphingolipid-triggered cell migration, respectively [75, 76]. Finally, Ser83 phosphorylation by calcium/CaMKII or PLK1 controls β1 integrin expression at the plasma membrane, controlling cell adhesiveness during invasion [77].

Importantly, phosphorylation of Ser39, Ser72 and Ser83 is preserved when vimentin is processed and peptides presented associated to major histocompatibility complexes. CD4^+^ T cells distinguish between non-phosphorylated *vs.* phosphorylated vimentin peptides [78]. Since these phosphorylations are elevated in metastatic cells [78], they could be useful as immunotherapeutic targets.

## Is it feasible to target cytoskeletal phosphorylation to treat cancer?

### A possible role for MT phosphorylation in cancer cell proliferation and migration

Tubulin phosphorylation is arguably the least understood of cytoskeletal phosphorylations. The apparent lack of interest in the field may be due to the fact that early use of anti-tubulin polymerization drugs such as colchicine or vincas; or MT turnover inhibitors, e.g., taxanes, was very successful to inhibit mitosis in cancer cells [79]. The scant information available emerges from global phospho-proteomics approaches. However, tubulin appears heavily phosphorylated in cancer cells, which strongly suggests that targeting phosphorylation could be of therapeutic interest. Two of the most prominent kinases that target MTs are AURKA and AURKB, and clinical approaches are underway to address the viability of their inhibition to treat different types of cancer [80]. However, the rationale behind their use is that AURK inhibition impairs mitosis, which is the same as targeting MT dynamics via vincas or taxanes. It is likely that AURK-dependent inhibition of cell division is independent of MT phosphorylation. Definitive proof would emerge from experiments aimed at interrogating whether phospho-mimetic forms of Ser48 (α-tubulin) and/or Ser75 (β-tubulin) confer resistance to AURK inhibitors. Recent work has highlighted that AURKA and PLK1 are essential for MT dynamics and centrosome positioning during T cell activation [81], hence their inhibition can play a role in curbing T-cell lymphomas and other T-cell-dependent malignancies. However, it will be important to determine whether the effect of AURK inhibitors is due to direct phosphorylation of tubulin.

An intriguing possibility is to target tubulin phosphorylation to complement vinca or taxane treatments, particularly to counteract the development of resistance, which is observed in many forms of advanced cancer. The assumption is that taxane-resistant cells develop mechanisms to undergo mitosis in the presence of these inhibitors, overcoming G2/M arrest, e.g., in the presence of hypoxia [82]. An earlier study showed that phosphorylation impairs tubulin polymerization promoting protein (TPPP)-dependent tubulin polymerization in the brain [83], confirming that targeting tubulin phosphorylation could affect its dynamics through different mechanisms than those of vinca or taxanes. In this manner, targeting tubulin phosphorylation could be a potentially useful approach to complement current therapies aimed at blocking cancer cell division.

Compared to their central role in cell division, the role of MTs in cell migration is more controversial. Thus, whether inhibiting their phosphorylation could have an effect on invasion is uncertain. MTs control cell polarity [84] and preserve the integrity of the cell as it migrates [85]. Since MTs control vesicle traffic, targeting MT dynamics through phosphorylation in this context could impair cancer cell secretion, decreasing the ability of cancer cells to exert modifications on the tumor microenvironment.

### Targeting myosin and actin phosphorylation: a gateway to curb metastasis

Actin and myosin are also important for mitosis. Different studies have shown that inhibiting MYH10 expression promotes multinucleation due to cytokinesis failure [86, 87]. Since NMII activation relies on RLC phosphorylation, it is theoretically possible to inhibit NMII function by targeting the kinases that mediate RLC Ser19 phosphorylation, which is critical for NMII filamentation. Multinucleation due to failed cytokinesis is also observed when myosin-specific kinases, e.g., CITK are inhibited or deleted [88]. CITK depletion, which reduces NMII phosphorylation and activation, reduces tumor growth in multiple myeloma [89] and medulloblastoma [90]. Interestingly, this does not happen when other myosin kinases, e.g., ROCK, are targeted (in fact, ROCK inhibitors are routinely used to culture stem cell *in vitro* to favor growth and prevent differentiation [91–93]). This highlights the central role of myosin regulation in many different processes, which may render targeting NMII phosphorylation impractical to inhibit proliferation.

On the other hand, targeting NMII (and actin) phosphorylation could prevent tumor cell dissemination. Different lines of evidence have suggested that elevated NMII phosphorylation and activity in the cortex changes the cellular phenotype from epithelial, or mesenchymal, into amoeboid [94, 95]. Amoeboid shape is characteristic of rapidly migrating cells, e.g., leukocytes, and is mainly integrin-independent [96]. In addition, elevated levels of phosphorylated NMII correlate with more aggressive tumors, e.g., gliosis-to-glioblastoma progression [97]. A recent study has highlighted that melanoma cells that undergo a mesenchymal-to-amoeboid transition display elevated levels of phosphorylated NMII, while also producing immunosuppressive signals [98]. The same group has shown that targeting ROCK alters the sensitivity of cancer cells to mitogen-activated protein kinase (MAPK) inhibitors [99], indicating that abnormalities in NMII phosphorylation downstream of ROCK may be targeted to confer cellular sensitivity to other families of inhibitors.

However, the different levels of regulation dependent on NMII phosphorylation are only beginning to be understood. This is a very active line of investigation in our lab.

### Possible effects of targeting vimentin phosphorylation in cancer cell division and tumor mechanics

Vimentin knockout mice are viable and fertile, displaying only minor developmental defects [100]. Other types of IFs are likely to compensate for the loss of vimentin in a tissue-specific manner, for example epidermal keratins in the skin or GFAP in the central nervous system [101].

Vimentin is prominently expressed in cells that are (or become) motile, including fibroblasts, mesenchymal cells, leukocytes and invasive cancer cells. This has suggested that vimentin could be targeted to prevent cancer cell motility. Indeed, vimentin is upregulated during EMT, which is a common occurrence in many carcinomas, and its repression in these cells decrease breast and colon tumor cell migration, as shown by siRNA depletion of vimentin in migrating mesenchymal cells and overexpression of vimentin in epithelioid, non-migrating tumor cells [102, 103]. In addition, higher expression of vimentin correlates with decreased survival in colorectal cancer, which could be related to a decreased metastatic ability [104].

Regarding vimentin phosphorylation, some sites favor IF assembly, whereas others promote disassembly, hence it is difficult to make general statements regarding the effect of its phosphorylation in cancer progression. An interesting fact is that many mitotic kinases, e.g., CDKs, PLK1, AURK, induce vimentin phosphorylation, hence its phosphorylation is likely to favor cell division. A model emerges in which elevated vimentin phosphorylation promotes IF disassembly, favoring cancer cell division. Likewise, recent evidence indicates that IFs may promote tumor cell migration [105], hence it is possible that similar mechanisms favor tumor cell dissemination. An intriguing possibility is that the preservation of these post-translational modifications during antigenic presentation [78] could be therapeutically useful to design novel immunotherapy-based strategies. Phospho-vimentin peptides could bear higher specificity for highly transformed cells, improving the cytotoxic T lymphocyte (CTL) response against them.

### Bystander inhibition of cytoskeletal phosphorylation in current therapies

While a few cytoskeletal components, e.g., myosin II, have well-defined kinomes, most of them lack specific kinases. However, most of the reported effects on actin, tubulin and vimentin phosphorylation in cancer cells emerge from studies using kinase-specific drugs. Hence, we cannot rule out that some specific phenotypes caused by current treatments are related to cytoskeletal phosphorylations. A key example is that of paclitaxel, which induces mitotic arrest at G2/M, promoting vimentin phosphorylation in Ser56 via CDK1 [71].

A few years ago, we demonstrated that dasatinib, a Tyr kinase inhibitor used to treat CML and Philadelphia chromosome-positive acute lymphoblastic leukemia (Ph^+^ALL) with several potential targets [106] induced myosin II phosphorylation, leading to increased contractility and vascular leakage [107]. This indicated that one of dasatinib substrates phosphorylated and inactivated a contractility inhibitor, perhaps ROCK. Likewise, we have recently shown that myosin light chain is a target of EGFR, which is a therapeutic target for the treatment of breast cancer, among others [37].

It is also possible that therapies aimed at inhibiting kinases in cancer cells have unexpected effects on non-cancer cells associated to the tumor microenvironment. For example, cancer-associated fibroblasts (CAFs) are very contractile, likely displaying elevated levels of phosphorylated NMII. NMII phosphorylation in these cells induces the formation of stress fibers, which predicts contact guidance for surrounding breast cancer cells [108]. Therapies designed to alter cytoskeletal phosphorylations in tumor cells could also have a dramatic impact on the tumor microenvironment, which will be a fascinating field of research in years to come.

## Conclusion

Targeting cytoskeletal phosphorylation has the potential to dramatically alter the mechano-chemical properties of tumor cells, inhibiting their ability to develop the cancer program, at least at a preclinical level. However, these approaches may also have potentially severe side effects, as every cell, normal or cancerous, requires the cytoskeleton. MT inhibitors, discovered over 60 years ago, offered early promise as MT-targeted therapies that improved the outcome of many types of cancer by inhibiting tumor cell division. These therapies are still the first line of chemotherapeutic treatment in many types of cancer. Hence, it is evident that phosphorylation of cytoskeletal components is altered when patients are treated with kinase-targeted therapies. We have only scratched the surface in characterizing these effects. Many of them will be unavoidable consequences of treatments aimed at other fundamental processes, potentially causing side effects that will have to be dealt with. But the potential exists to discover that some cytoskeletal phosphorylation-specific effects underlie unexpected and important effects of current and future targeted therapies.

## Abbreviations

ATP: adenosine triphosphate
AURK: Aurora kinase
BCR: breakpoint cluster region
CDK: cyclin dependent kinase
CITK: citron kinase
EGFR: epidermal growth factor receptor
ELC: essential light chain
EMT: epithelial-mesenchymal transition
ERK: extracellular signal-regulated kinase
IF: intermediate filament
MEK: mitogen-activated protein kinase kinase
MF: microfilament
MHCII: myosin heavy chain II
MRCK: myotonic dystrophy kinase-related
MT: microtubule
NMII: non-muscle myosin II
PAK1: p21-activated kinase 1
PKC: protein kinase C
PLK1: polo-like kinase 1
RLC: regulatory light chain
ROCK: RhoA-coiled coil kinase
SadB: synapses of amphids defective kinase
Ser: serine
Thr: threonine
TRPM6/7: transient receptor potential melastatin 6/7
Tyr: Tyrosine
ZIPK: zipper-interacting protein kinase

## Supplementary materials

The supplementary materials for this article are available at: https://www.explorationpub.com/uploads/Article/file/100247_sup_1.pdf.

## Declarations

### Availability of data and materials

Data are publicly available and reproduced from public and private, but fully and freely searchable, databases.

### Copyright

© The Author(s) 2021.
