# Supplementary material for: Targeting cytoskeletal phosphorylation in cancer
Source: Explor Target Antitumor Ther. 2021 Jun 28;2(3):292–308. doi: 10.37349/etat.2021.00047 (PMC9400739; doi:10.37349/etat.2021.00047)
Supplement: Supplementary file 1 [file etat-02-100247-s001.pdf]

**Table S1.** Full homology among human tubulin isoforms

| | | TUBA1A | TUBA1B | TUBA1C | TUBA3C | TUBA3D | TUBA3E | TUBB | TUBB1 | TUBB2A | TUBB2B | TUBB3 | TUBB4A | TUBB4B | TUBB6 | TUBG1 | TUBG2 |
|---|---|---|---|---|---|---|---|---|---|---|---|---|---|---|---|---|---|
| TUBA1A | NP_006000.2 | 100 | 99.56 | 98 | 97.56 | 97.56 | 96.23 | 39.55 | 40 | 40.32 | 40.32 | 40.71 | 40.55 | 40.78 | 41.24 | 31.52 | 31.52 |
| TUBA1B | NP_006073.2 | | 100 | 98 | 97.12 | 97.12 | 96.59 | 40 | 40.46 | 40.78 | 40.78 | 41.15 | 41.01 | 41.24 | 41.71 | 31.75 | 31.75 |
| TUBA1C | NP_116093.1 | | | 100 | 96.22 | 96.22 | 96.13 | 39.77 | 39.77 | 40.55 | 40.55 | 40.98 | 40.78 | 41.01 | 41.47 | 31.75 | 31.75 |
| TUBA3C | NP_005992.1 | | | | 100 | 100 | 98.67 | 39.32 | 39.77 | 40.09 | 40.09 | 40.44 | 40.32 | 40.55 | 41.01 | 31.07 | 31.07 |
| TUBA3D | NP_525125.2 | | | | | 100 | 98.67 | 39.32 | 39.77 | 40.09 | 40.09 | 40.44 | 40.32 | 40.55 | 41.01 | 31.07 | 31.07 |
| TUBA3E | NP_997195.2 | | | | | | 100 | 38.86 | 39.86 | 39.63 | 40.19 | 40 | 39.86 | 40.09 | 40.55 | 30.99 | 30.99 |
| TUBB | NP_001280141.1 | | | | | | | 100 | 79.37 | 96.84 | 97.33 | 92.25 | 96.94 | 97.65 | 92.03 | 31.81 | 32.7 |

| | | | | | | | | | | |
|---|---|---|---|---|---|---|---|---|---|---|
| TUBB1 | NP_110400.1 | 100 | 80.7 | 80.93 | 78.22 | 80.33 | 80.33 | 80 | 34.4 | 34.4 |
| TUBB2A | NP_001060.1 | | 100 | 99.55 | 91.93 | 96.25 | 97.66 | 92.13 | 34.17 | 34.17 |
| TUBB2B | NP_821080.1 | | | 100 | 92.15 | 96.72 | 96.85 | 92.36 | 34.17 | 34.17 |
| TUBB3 | NP_006077.2 | | | | 100 | 93.21 | 93.91 | 93.98 | 34.4 | 34.4 |
| TUBB4A | NP_006078.2 | | | | | 100 | 98.43 | 93.07 | 34.17 | 34.17 |
| TUBB4B | NP_006079.1 | | | | | | 100 | 97 | 34.62 | 34.62 |
| TUBB6 | NP_115914.1 | | | | | | | 100 | 33.71 | 33.94 |
| TUBG1 | NP_001061.2 | | | | | | | | 100 | 100 |
| TUBG2 | NP_057521.1 | | | | | | | | | 100 |
